# A survival analysis of COVID-19 in the Mexican population

**DOI:** 10.1186/s12889-020-09721-2

**Published:** 2020-10-27

**Authors:** Guillermo Salinas-Escudero, María Fernanda Carrillo-Vega, Víctor Granados-García, Silvia Martínez-Valverde, Filiberto Toledano-Toledano, Juan Garduño-Espinosa

**Affiliations:** 1 Center for Economic and Social Studies in Health, Hospital Infantil de México Federico Gómez, Mexico City, Mexico; 2 Geriatric Epidemiology Unit, Research Department, Instituto Nacional de Geriatría, Av. Contreras 428, Col. San Jerónimo Lídice, Alcaldía Magdalena Contreras, Mexico City, Mexico; 3 Epidemiological and Health Services Research Unit Aging Area, Centro Médico Nacional, Siglo XXI, Mexico City, Mexico; 4 Research Unit in Evidence-Based Medicine, Hospital Infantil de México Federico Gómez, Mexico City, Mexico; 5 Research Department, Hospital Infantil de México Federico Gómez, Mexico City, Mexico

**Keywords:** COVID-19, Pandemics, Mortality, Risk factors

## Abstract

**Background:**

At present, the Americas report the largest number of cases of COVID-19 worldwide. In this region, Mexico is the third country with most deaths (20,781 total deaths). A sum that may be explained by the high proportion of people over 50 and the high rate of chronic diseases. The aim of this analysis is to investigate the risk factors associated with COVID-19 deaths in Mexican population using survival analysis.

**Methods:**

Our analysis includes all confirmed COVID-19 cases contained in the dataset published by the Epidemiological Surveillance System for Viral Respiratory Diseases of the Mexican Ministry of Health. We applied survival analysis to investigate the impact of COVID-19 on the Mexican population. From this analysis, we plotted Kaplan-Meier curves, and constructed a Cox proportional hazard model.

**Results:**

The analysis included the register of 16,752 confirmed cases of COVID-19 with mean age 46.55 ± 15.55 years; 58.02% (*n* = 9719) men, and 9.37% (*n* = 1569) deaths. Male sex, older age, chronic kidney disease, pneumonia, hospitalization, intensive care unit admission, intubation, and health care in public health services, were independent factors increasing the risk of death due to COVID-19 (*p* < 0.001).

**Conclusions:**

The risk of dying at any time during follow-up was clearly higher for men, individuals in older age groups, people with chronic kidney disease, and people hospitalized in public health services.

**Supplementary Information:**

The online version contains supplementary material available at 10.1186/s12889-020-09721-2.

## Background

The pandemic caused by the novel coronavirus, SARS-CoV-2, has become one of the biggest health challenges worldwide. There are nearly 9 million confirmed cases and more than 465 thousand deaths as of June 22nd, 2020. This makes the COVID-19 pandemic one of the public health problems with a meaningful impact in the history of humanity.

By the third week of June, the Americas were reporting the largest number of cases worldwide. The United States was the country with the highest number of total confirmed COVID-19 cases (2,241,178), followed by Brazil (1,067,579 cases), Perú (251,338 cases), Chile (242,355 cases), and Mexico (175,202 cases). However, Mexico was third in the death count (20,781 total deaths) [1].

COVID-19 is commonly asymptomatic [2]. Asymptomatic COVID-19 patients frequently improve with time without specialized medical care [3]. But a significant proportion of COVID-19 cases develop pneumonia and acute severe respiratory failure [4]. These cases commonly require hospitalization, intensive care unit admission, and intubation [5].

Older age, the presence of comorbidities, particularly hypertension, diabetes, obesity, and smoking are factors that increase the risk of severe disease [6, 7]. The characteristics of COVID-19 may vary depending on the demographic and epidemiological profiles of each country. In the specific case of Mexico, 27% of the population is over 50 years old [8] and there is a high rate of chronic diseases [9, 10] which may increase the risk of fatal complications.

Assessing the instantaneous rate of death at any time during follow-up for the specific risk factors is crucial to determine the appropriateness of the mitigation strategies and to set up priorities to control the COVID-19 epidemic. This is especially so for countries that are in the present moment focusing their efforts on fighting the pandemic; this is the case of Mexico. The present analysis aimed to investigate the risk factors associated with COVID-19 deaths in the Mexican population using survival analysis.

## Methods

### Design and settings

The data released by the Mexican Ministry of Health (Secretaría de Salud, SS) through the Epidemiological Surveillance System for Viral Respiratory Diseases on suspected viral respiratory disease cases were used for the present analysis [11]. The database included all positive, negative, and suspected cases of COVID-19 registered by 475 Viral Respiratory Disease Monitoring Units (Unidades Monitoras de Enfermedad Respiratoria viral; USMER by its Spanish acronym) and by the medical units that attended the cases.

The information recorded on every individual includes: sex, age, nationality, place of residence, migratory status, chronic diseases, immune-suppression, and other diseases reported by the individual, smoking and pregnancy. Data registered on the COVID-19 event includes: type of first contact medical unit (SS or private services), management received (either hospitalization or outpatient), and dates of onset of COVID-19 symptoms, admission to hospitalization, development of pneumonia, admission to intensive care units (ICU), intubation, and death. Data on the evolution during the stay in the medical units were not released for public use.

All the confirmed cases of COVID-19 registered from February 21st to April 28th, 2020, were used in the present analysis. Eleven records were eliminated as the onset of symptoms and the time of death were registered with the same date. The original dataset included seven cases with symptoms starting on February 21st, 7 days before the first case was officially reported. Thus, these registers were eliminated from the survival analysis.

### Variables

The outcome variable was time to death, constructed as the time between date of symptoms onset and death (failure) with censoring on April 28th, 2020 for individuals who were alive by the end of the study period.

Our analysis considers the following variables: age, sex, comorbidities, pregnancy, immune-suppression, smoking, time elapsed between the onset of symptoms and hospitalization, and death, as well as the time elapsed from admission to health care unit to death, development of pneumonia, hospitalization, ICU admissions, intubation, and the type of health service.

The survival analysis and the Cox proportional hazard model included dichotomic values for sex, morbidity (yes or no for each disease), pneumonia, hospitalization, ICU, and intubation. We grouped health services into 5 categories: IMSS, ISSSTE, SS, Other Public Services, and Private Services. We grouped age in four tiers: < 25 years old, 25–49 years old, 50–74 years old, and ≥ 75 years old.

#### Statistical analysis

This paper describes continuous variables using means and standard deviations (±SD), and categorical variables expressed as number and percentage (%). The mortality rate per 1000 person-years was calculated for the general sample and both sexes. Histograms were graphed to assess the symmetry in the dispersion of the values to select the best test to use. In addition, the bias-kurtosis and Shapiro-Wilk tests were used to evaluate the normality of the covariates and select the statistical tests. Since no variable had a normal distribution, comparisons between individuals who died versus those who survived, were made through the Mann-Whitney test for counting variables, and x^2^ for categorical variables.

The Kaplan-Meier method was used to plot survival curves. These graphs served to test the proportional hazard assumption. We fitted a Cox’s Proportional Hazards Model including the covariables of interest using a step forward process, i.e. from a null model, all the covariates with *p* < 0.05. were included in the model. The data was analyzed with the statistical package software Stata version 14.0 (StataCorp, College Station, Texas, USA).

## Results

This analysis includes 16,752 registers of confirmed cases of COVID-19. The mean age of the individuals was 46.55 ± 15.55 years, 58.02% (*n* = 9719) were men, and 9.37% (*n* = 1569) died. The general mortality rate was 4.94 (95% CI 4.70–5.19) per 1000 person-year, for men 5.82 (95% CI 5.48–6.18) per 1000 person-year, and for women 3.74 (95% CI 3.43–4.08) per 1000 person-year (Table 1).

**Table 1:** General characteristics by outcome

	Total *n* = 16,752	Dead *n =* 1569	Alive *n* = 15,183	*p*-value
Age, years	46.55 ± 15.55	59.42 ± 14.29	45.22 ± 15.06	< 0.001 ^a^
Sex (men)	9719 (58.02)	1066 (67.94)	8653 (56.99)	< 0.001 ^b^
Mortality rate (95% CI)^c^		4.94 (4.70–5.19)		
Men (95% CI)^c^		5.82 (5.48–6.18)		
Women (95% CI)^c^		3.74 (3.43–4.08)		
Comorbidities				
Diabetes	3064 (18.44)	266 (17.12)	2798 (18.58)	0.158 ^b^
COPD	421 (2.53)	44 (2.83)	377 (2.5)	0.435 ^b^
Asthma	585 (3.52)	51 (3,.28)	534 (3.55)	0.588 ^b^
Hypertension	3640 (21.91)	353 (22.72)	3287 (21.82)	0.418 ^b^
Cardiovascular disease	473 (2.85)	43 (2.77)	430 (2.86)	0.878 ^b^
Obesity	3463 (20.83)	340 (21.86)	3123 (20.73)	0.293 ^b^
CKD	388 (2.34)	107 (6.86)	281 (1.87)	< 0.001 ^b^
Number of comorbidities	0.72 ± 0.94	0.77 ± 0.95	0.71 ± 0.94	0.029 ^a^
No comorbidities	9173 (54.76)	417 (26.58)	8756 (57.67)	< 0.001 ^b^
Pregnancy	99 (0.59)	5 (0.32)	95 (0.62)	0.138 ^b^
Immune-suppression	314 (1.89)	25 (1.61)	289 (1.92)	0.391 ^b^
Smoking	1496 (9)	150 (9.62)	1346 (8.94)	0.368 ^b^
Onset of symptoms-admission (days)	4.27 ± 3.43	4.29 ± 3.49	4.26 ± 3.42	0.730 ^a^
Onset of admission-dead (days)	5.86 ± 5.12	5.86 ± 5.12	–	
Onset of symptoms-dead (days)	10.15 ± 5.75	10.15 ± 5.75	–	
Pneumonia	4942 (29.5)	1182 (75.33)	3760 (24.76)	< 0.001 ^b^
Hospitalized	6581 (39.28)	1416 (90.25)	5165 (34.02)	< 0.001 ^b^
ICU	720 (4.3)	292 (18.61)	428 (2.82)	< 0.001 ^b^
Intubated	709 (4.23)	376 (23.96)	333 (2.19)	< 0.001 ^b^
IMSS services	6262 (37.57)	667 (42.65)	5595 (37.04)	< 0.001 ^b^
ISSSTE services	905 (5.43)	124 (7.93)	781 (5.17)	< 0.001 ^b^
SS services	7896 (47.37)	655 (41.88)	7241 (47.94)	< 0.001 ^b^
Other public services	684 (4.1)	87 (5.56)	597 (3.95)	0.002 ^b^
Private services	922 (5.53)	31 (1.98)	891 (5.9)	< 0.001 ^b^

^a^Mann-Whitney test

^b^χ^2^ test

^c^Mortality rate per 1000 person-year

*COPD* Chronic Obstructive Pulmonary Disease, *CKD* Chronic Kidney Disease, *ICU* Intensive Care Unit, *IMSS* Mexican Institute of Social Services, *ISSSTE* Institute of Social Security and Services for State Workers, *SS* Mexican Ministry of Health

When comparing individuals who died versus those who survived, the deceased were older (59.42 ± 14.29) and mostly men (67.94%), (*p* < 0.001). The prevalence of CKD in people who died (6.86%) was larger in comparison to those who did not die (*p* < 0.001). In individuals who survived, the proportion without comorbidities was higher (*p <* 0.001). The period from the onset of symptoms to admission in hospitalization was similar between both groups (4.27 ± 3.43, *p* = 0.7302). The period from the onset of symptoms to death, and the period from admission to death were 10.15 ± 5.75 and 5.86 ± 5.12 days, respectively. Among the people who died 75.33% (*n* = 1182) developed pneumonia, 90.25% (1416) was hospitalized, 18.61% (*n* = 292) was admitted to the ICU, and 23.96% (*n* = 376) needed intubation. These proportions were higher in comparison to individuals who did not die (*p* < 0.001). In the group of those who died, 42.65% received health care from IMSS, while in the surviving group, 47.94% received health care from SS services (Table 1).

**Fig. 1:**
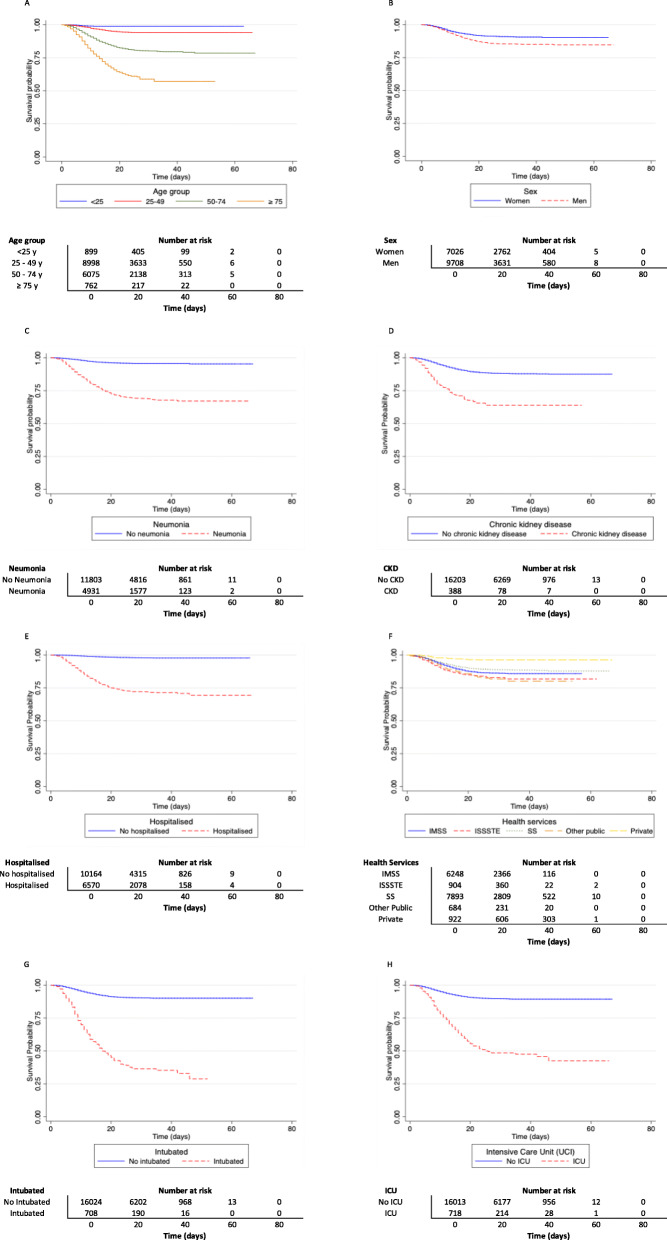
Kaplan-Meier survival plots for different prognostic factors

The survival analysis comprises a total of 16,734 registers, 1558 of whom died. Our analysis observed a cumulated total risk time of 315,773 days. The Kaplan-Meier survival plots for the prognostic factors that resulted statistically significant are presented in Fig. 1. As can be inferred from the plot, risk is directly proportional to age group. The proportional hazards assumption is satisfied since survival risk curves do not cross during the analyzed period. Individuals who developed pneumonia, those with a previous diagnosis of CKD, and those who were admitted to hospitalization, have approximately 30% lower probability of survival after 20 days of hospitalization than individuals who did not present these characteristics. People who were admitted to ICU, have about 40% less probability of survival after 20 days of hospitalization than individuals who did not enter this unit. Finally, individuals who needed intubation have just about 50% probability of survival after 20 days of hospitalization compared to individuals who were not intubated.

The Kaplan-Meier plots demonstrated that the risk of mortality was different between sexes. Thus, Kaplan-Meier curves were plotted for men and women (Supplementary material 1 and 2), and multivariable Cox proportional hazards regression models were run for each sex. The hazard ratios for both sexes can be seen in Table 2. For women, the multivariable model containing the significant covariates was statistically significant (x^2^ = 22.22, df = 10, *p* < 0.01). At any time during follow-up, age increases the risk of dying, and that risk was significantly high in older women, who had 4.41 times the risk of dying compared with the youngest age group. Women with CKD were 1.90 times likely to die compared with women without this disease. Twice as many women who developed pneumonia had died compared to women who did not develop this complication. At any time during the follow-up, women admitted to hospitalization were 6.57 times as likely to die compared with those who were not admitted to a hospital. Women who needed intubation were 2.83 times as likely to die compared with those who did not require intubation. Women receiving health care from IMSS services had 4.34 times the risk of dying compared to women who received health care in private facilities.

**Table 2:** Hazard ratio for the fatal outcome in women and men

	Women				Men			
	Hazard Ratio	*p*-value	95% CI for the Hazard Ratio		Hazard Ratio	*p*-value	95% CI for the Hazard Ratio	
Age ^a^								
25–49 y					4.85	0.007	1.55	15.13
50–74 y	2.37	*p <* 0.001	1.88	2.99	8.71	*p <* 0.001	2.79	27.13
75 + y	4.41	*p <* 0.001	3.32	5.87	15.46	*p <* 0.001	4.91	48.70
Chronic kidney disease	1.90	*p <* 0.001	1.36	2.66	1.79	*p <* 0.001	1.40	2.30
Pneumonia	2.09	*p <* 0.001	1.63	2.67	2.05	*p <* 0.001	1.74	2.42
Intubation	2.83	*p <* 0.001	2.20	3.63	1.39	0.001	1.15	1.69
Hospitalization	6.57	*p <* 0.001	4.69	9.22	3.12	*p <* 0.001	2.57	3.79
Health Services (Private, reference)					5.42	*p <* 0.001	4.25	6.91
IMSS Services	4.34	*p <* 0.001	2.28	8.26				
ISSSTE Services	3.12	0.001	1.57	6.18	6.35	*p <* 0.001	4.06	9.92
SS Services	3.22	*p <* 0.001	1.70	6.08	2.85	*p <* 0.001	1.75	4.64
Other Public services	2.76	0.006	1.35	5.65	3.52	*p <* 0.001	2.27	5.47

^a^Women reference group ≤49 years, Men reference group ≤24 years

*IMSS* Mexican Institute of Social Services, *ISSSTE* Institute of Social Security and Services for State Workers, *SS* Mexican Ministry of Health

The multivariable Cox proportional hazards regression model for men was statistically significant (χ^2^ = 31.83, df = 12, *p* < 0.001) and is presented in Table 2. The risk of dying was notably higher for men over 75 years old. They had 15.46 times the risk of dying compared with the youngest age group. Men with CKD were 1.79 times more likely to die compared with men without this disease. Pneumonia and intubation multiplied by 2.05 and 3.12 respectively the risk of dying for men. Men admitted to hospitalization and ICU were 5.42 and 1.39 times as likely to die, respectively, compared with those who did not enter these units. At any time during follow-up, men who needed intubation were 3.12 times as likely to die compared with those who did not require intubation. Men who received health care from IMSS had 6.35 times the risk of dying compared to those who received health care in private facilities.

## Discussion

The results of our analysis, with the Kaplan-Meier survival method and multivariable Cox proportional hazards regression model, demonstrated that men had a higher risk of dying due to COVID-19. In both sexes, older age, CKD, development of pneumonia, hospitalization, intubation, and health care in public health services are independent risk factors increasing the risk of death due to COVID-19. ICU admission was a significant risk factor only in men.

The systematic review and meta-analysis from Galbadage et al. [12] showed that men are more likely to die from COVID-19 than women. Although a clear explanation of this association has not been established yet, it has been suggested that some biological and immunological pathways can play an essential role in the differential behavior of COVID-19 [13]. Some socio-behavioral and cultural aspects can also explain the more unfavorable scenario for men [14, 15]. It has been reported that being over 50 years old increases the risk of fatal complications for this disease [16, 17]. Both sex and old age play an essential role in the risk of the Mexican population. It is especially striking that men in the oldest age group have about 16 times the risk of dying from COVID-19.

ICU admission and intubation are indicators of high-level severity of the disease, both represent an increased risk of death [18]. In our analysis, intubation represents a notable risk factor since it more than doubles the risk of death in both men and women.

The mean time from the onset symptoms to death (Table 1) was different from that reported in other studies suggesting that death occurs between 14 and 21 days after the onset of symptoms [19]. We found that the time between the onset of symptoms until seeking care was similar between people who died and those who survived (*p* = 0.7302). It can be hypothesized that people with a high risk of severe COVID-19, e.g. with comorbidities, arrived in a critical state to the health services.

A high proportion (58%) of people who survived COVID-19 had no comorbidities. The high prevalence of chronic diseases, in both individuals who died and those who did not, reflects the epidemiological context of Mexico [9, 10] but also the impact of chronic disease in the COVID-19 presentation and severity. These data allows us to hypothesize that chronic diseases may not only increase the risk of complicated disease but also the risk of acquiring the novel coronavirus. A possible explanation is that chronic inflammation, adverse effects on immunomodulation, and metabolic stress that characterize systemic diseases, decrease the ability to react against external agents; in this case, SARS-CoV-2 [20–22].

In our analysis, CKD a common comorbidity of diabetes [23] and hypertension [24], increased almost two times the risk of death in comparison to people without this disease. Cheng et al. [25] reported that 13.1% of the people admitted to hospitalization in China had kidney failure. This prevalence is significantly larger than the 2.34% we found in the Mexican population diagnosed with COVID-19. Besides, in the Chinese study, the mortality rate due to CKD (33%) was more extensive than in our population. While in China, kidney disease was diagnosed at the hospital admission by laboratory tests, in Mexico, the diagnosis was registered on a self-reported basis. Thus, the prevalence and mortality due to this cause may be underestimated in Mexico. The Kaplan-Meier curves in the Chinese study also show that individuals with kidney disease had a significantly higher risk of in-hospital death. A similar pattern can be observed in the present study. It has been suggested that SARS-CoV-2 may bind to renal epithelial cells, injure these cells, and subsequently disrupt the whole body fluid, acid-base, and electrolyte homeostasis, thus increasing the failure in those with preexisting CKD therefore accelerating their deaths [26, 27].

A relevant finding of our analysis is that the crude lethality rate of 9.4% is above the 5.3% initially reported in Hubei, China [28], and 6.05% in the United States. However, it is below the 11.76% for Spain, 13.98% for Italy, 14.37% for the United Kingdom and 19.35% for France [29]. Such discrepancies can be explained by the way the COVID-19 cases are confirmed and deaths registered. In most countries, only in-hospital deaths are recorded, and in countries like Mexico, in the absence of symptoms, tests are not used in large quantities [30].

Finally, according to our results, different health service providers may have an essential role in lethality. Public providers reported a larger risk of death in comparison with private ones. IMSS presents the highest risk of death (hazard ratio 4.34 in women and 6.35 in men), followed by SS services (hazard ratio 3.22 and 3.52 in women and men, respectively). It is especially important to note that SS and IMSS are the institutions that have received the most cases of COVID-19 in this epidemic, both of uninsured and insured population, hence, having a high probability of over saturation.

The analysis of these results must consider some limitations. First, the dataset does not include clinical variables related to the evolution of the COVID-19, which could have been useful in adjusting the model. Second, since the COVID-19 pandemic is ongoing, not all outcomes of the pandemic have been observed. Finally, The database contains information on all deaths that were confirmed as positive cases of COVID-19. Nevertheless, there is a high percentage of deaths classified as atypical pneumonia which were not confirmed as cases of COVID-19. Those cases may lead to underestimate the case fatality rate for this disease.

Nonetheless, the present paper contributes to understand the dynamics of the pandemic in Mexico. We highlight risk factors for death that have been little studied, especially the presence of CKD and data about the health system that reflects the social inequality in Mexico.

## Conclusions

This analysis adds to previous evidence [31] on the higher risk of death for men and older people, both in Mexico and elsewhere. Further research is necessary to understand the origin of this differential risk. One of the specific goals of the basic and clinical research should be to identify differences in response to vaccination to ensure the efficacy of the novel vaccines.

We consider that other risk factors will become relevant over time, so epidemiological research will continue to be paramount.

Given the persistence of SARS-Cov-2, it is vital to focus mitigation campaigns on the population at higher risk of fatal outcomes. We also need stronger public health campaigns aimed at reducing the prevalence of obesity, diabetes, hypertension, and their comorbidities. These conditions may increase the susceptibility of the Mexican population to COVID-19 and cause a rapid progression to severe states of the disease and death.

## Supplementary information

**Additional file 1: Fig. S1.** Kaplan-Meier survival plots for different prognostic factors for women. The figure displays the Kaplan-Meier survival plots according to (A) age, (B) neumonia, (C) CKD, (D) Hospitalized, (E) Health Services, (F) Intubated and (G) Intensive Care Unit. **Fig. S2.** Kaplan-Meier survival plots for different prognostic factors for men. The figure displays the Kaplan-Meier survival plots according to (A) age, (B) neumonia, (C) CKD, (D) Hospitalized, (E) Health Services, (F) Intubated and (G) Intensive Care Unit.

## Data Availability

The dataset analyzed during the current study is available in the Mexican Ministry of Health repository, https://www.gob.mx/salud/documentos/datos-abiertos-152127

## Abbreviations

CKD: Chronic kidney disease
COPD: Chronic obstructive pulmonary disease
COVID-19: Coronavirus disease 2019
ICU: Intensive care units
IMSS: Mexican Institute of Social Security
ISSSTE: Institute of Social Security and Services of the State Workers
SARS-CoV-2: Severe acute respiratory syndrome coronavirus 2
SS: Mexican Ministry of Health
USMER: Viral Respiratory Disease Monitoring Units
