# Correction to: A survival analysis of COVID-19 in the Mexican population

**DOI:** 10.1186/s12889-020-09934-5

**Published:** 2020-11-30

**Authors:** Guillermo Salinas-Escudero, María Fernanda Carrillo-Vega, Víctor Granados-García, Silvia Martínez-Valverde, Filiberto Toledano-Toledano, Juan Garduño-Espinosa

**Affiliations:** 1 Center for Economic and Social Studies in Health, Hospital Infantil de México Federico Gómez, Mexico City, Mexico; 2 Geriatric Epidemiology Unit, Research Department, Instituto Nacional de Geriatría, Av. Contreras 428, Col. San Jerónimo Lídice, Alcaldía Magdalena Contreras, Mexico City, Mexico; 3 Epidemiological and Health Services Research Unit Aging Area, Centro Médico Nacional, Siglo XXI, Mexico City, Mexico; 4 Research Unit in Evidence-Based Medicine, Hospital Infantil de México Federico Gómez, Mexico City, Mexico; 5 Research Department, Hospital Infantil de México Federico Gómez, Mexico City, Mexico

**Correction to: BMC Public Health (2020) 20:1616**

**https://doi.org/10.1186/s12889-020-09721-2**

It was highlighted that in the original article [1] the letter χ was missing in footnote b of Table 1 and on the equation in the Results section on page 5. This Correction article shows the correct Table 1 footnotes and the correct equation.

**Table 1:** General characteristics by outcome

	Total n = 16,752	Dead n = 1569	Alive n = 15,183	p-value
Age, years	46.55 ± 15.55	59.42 ± 14.29	45.22 ± 15.06	< 0.001 ^a^
Sex (men)	9719 (58.02)	1066 (67.94)	8653 (56.99)	< 0.001 ^b^
Mortality rate (95% CI)^c^		4.94 (4.70–5.19)		
Men (95% CI)^c^		5.82 (5.48–6.18)		
Women (95% CI)^c^		3.74 (3.43–4.08)		
Comorbidities				
Diabetes	3064 (18.44)	266 (17.12)	2798 (18.58)	0.158 ^b^
COPD	421 (2.53)	44 (2.83)	377 (2.5)	0.435 ^b^
Asthma	585 (3.52)	51 (3,.28)	534 (3.55)	0.588 ^b^
Hypertension	3640 (21.91)	353 (22.72)	3287 (21.82)	0.418 ^b^
Cardiovascular disease	473 (2.85)	43 (2.77)	430 (2.86)	0.878 ^b^
Obesity	3463 (20.83)	340 (21.86)	3123 (20.73)	0.293 ^b^
CKD	388 (2.34)	107 (6.86)	281 (1.87)	< 0.001 ^b^
Number of comorbidities	0.72 ± 0.94	0.77 ± 0.95	0.71 ± 0.94	0.029 ^a^
No comorbidities	9173 (54.76)	417 (26.58)	8756 (57.67)	< 0.001 ^b^
Pregnancy	99 (0.59)	5 (0.32)	95 (0.62)	0.138 ^b^
Immune-suppression	314 (1.89)	25 (1.61)	289 (1.92)	0.391 ^b^
Smoking	1496 (9)	150 (9.62)	1346 (8.94)	0.368 ^b^
Onset of symptoms-admission (days)	4.27 ± 3.43	4.29 ± 3.49	4.26 ± 3.42	0.730 ^a^
Onset of admission-dead (days)	5.86 ± 5.12	5.86 ± 5.12	–	
Onset of symptoms-dead (days)	10.15 ± 5.75	10.15 ± 5.75	–	
Pneumonia	4942 (29.5)	1182 (75.33)	3760 (24.76)	< 0.001 ^b^
Hospitalized	6581 (39.28)	1416 (90.25)	5165 (34.02)	< 0.001 ^b^
ICU	720 (4.3)	292 (18.61)	428 (2.82)	< 0.001 ^b^
Intubated	709 (4.23)	376 (23.96)	333 (2.19)	< 0.001 ^b^
IMSS services	6262 (37.57)	667 (42.65)	5595 (37.04)	< 0.001 ^b^
ISSSTE services	905 (5.43)	124 (7.93)	781 (5.17)	< 0.001 ^b^
SS services	7896 (47.37)	655 (41.88)	7241 (47.94)	< 0.001 ^b^
Other public services	684 (4.1)	87 (5.56)	597 (3.95)	0.002 ^b^
Private services	922 (5.53)	31 (1.98)	891 (5.9)	< 0.001 ^b^

^a^ Mann-Whitney test; ^b^ χ^2^ test; ^c^ Mortality rate per 1000 person-year

COPD = Chronic Obstructive Pulmonary Disease; CKD = Chronic Kidney Disease; ICU = Intensive Care Unit; IMSS Mexican Institute of Social Services; ISSSTE Institute of Social Security and Services for State Workers; SS = Mexican Ministry of Health

**Correct** Table 1**footnotes**

^a^ Mann-Whitney test

^b^ χ^2^ test

^c^ Mortality rate per 1000 person-year

COPD Chronic Obstructive Pulmonary Disease, CKD Chronic Kidney Disease, ICU Intensive Care Unit, IMSS Mexican Institute of Social Services, ISSSTE Institute of Social Security and Services for State Workers, SS Mexican Ministry of Health

**Correct equation in Results section**

(χ^2^ = 31.83, df = 12, p < 0.001)

The original article has been updated.
