# Supplementary material for: A survival analysis of COVID-19 in the Mexican population
Source: BMC Public Health. 2020 Oct 27;20:1616. doi: 10.1186/s12889-020-09721-2 (PMC7588954; doi:10.1186/s12889-020-09721-2)
Supplement: Supplementary file 1 — Additional file 1: Fig. S1. Kaplan-Meier survival plots for different prognostic factors for women. The figure displays the Kaplan-Meier survival plots according to (A) age, (B) neumonia, (C) CKD, (D) Hospitalized, (E) Health Services, (F) Intubated and (G) Intensive Care Unit. Fig. S2. Kaplan-Meier survival plots for different prognostic factors for men. The figure displays the Kaplan-Meier survival plots according to (A) age, (B) neumonia, (C) CKD, (D) Hospitalized, (E) Health Services, (F) Intubated and (G) Intensive Care Unit. [file 12889_2020_9721_MOESM1_ESM.docx]

**Supplementary Material**

Figure 1. Kaplan-Meier survival plots for different prognostic factors for women. The figure displays the Kaplan-Meier survival plots according to (A) age, (B)neumonia, (C) CKD, (D) Hospitalised, (E) Health Services, (F) Intubated and (G) Intensive Care Unit.

Figure 2. Kaplan-Meier survival plots for different prognostic factors for men. The figure displays the Kaplan-Meier survival plots according to (A) age, (B)neumonia, (C) CKD, (D) Hospitalised, (E) Health Services, (F) Intubated and (G) Intensive Care Unit.
